# Exploring the diversity of *Ralstonia solanacearum* strains from mainland France using a novel multiple loci VNTR analysis scheme

**DOI:** 10.1128/aem.00963-25

**Published:** 2025-07-31

**Authors:** Antinéa Sallen, Aurélie Leclerc, Sandrine Paillard, Charles Poncet, Philippe Reignault, Anne-Claire Le Roux, Amandine Cunty

**Affiliations:** 1 Plant Health Laboratory, ANSES, Angers, France; 2 inov3PT, Paris, France; 3 IGEPP, INRAE, Institut Agro, Univ Rennes 27057 , Le Rheu, France; 4 GDEC, INRAE, UCA, Clermont-Ferrand, France; UMR Processus Infectieux en Milieu Insulaire Tropical, Ste. Clotilde, France

**Keywords:** *Ralstonia solanacearum*, mainland France, diversity, genotyping, MLVA

## Abstract

The *Ralstonia solanacearum* species complex (RSSC), one of the most damaging pests worldwide, includes species *Ralstonia solanacearum*, *Ralstonia pseudosolanacearum*, and *Ralstonia syzygii*. Since 1990, *R. solanacearum* has been reported in several European countries, including France. However, the genetic diversity of *R. solanacearum* in mainland France remains poorly characterized. In this study, a novel 14-variable number of tandem repeats multilocus variable-number tandem-repeat analysis (MLVA) scheme specifically designed for IIB-1 strains of *R. solanacearum* is presented. This scheme effectively differentiated 384 global strains, isolated between 1963 and 2023, into 66 distinct haplotypes. The near clonality of the IIB-1 genetic group was highlighted by the presence of a predominant clonal complex encompassing 82% of the haplotypes. The 346 strains from mainland France used in this study, isolated between 1994 and 2023, were differentiated into 46 haplotypes, which suggests that, while French *R. solanacearum* IIB-1 strains have been isolated from a relatively confined geographical area over time, they still exhibit some level of genetic diversity. Moreover, this MLVA scheme enabled clear discrimination between global and French/European strains which did not cluster together, allowing the inference of potential invasion routes for certain haplotypes in Europe. This MLVA scheme is thus a valuable tool for epidemiological studies, providing deeper insights into the genetic diversity of global IIB-1 strains.

**IMPORTANCE:**

The RSSC causes bacterial wilt on over 250 plant species, including major ones like potato, and includes species *R. solanacearum*, *R. pseudosolanacearum*, and *R. syzygii*, all characterized as quarantine organisms in the European Union. *R. solanacearum* has been reported in several European countries over the last century with major outbreaks confirmed to be IIB-1 strains in the 1990s, and recent outbreaks of *R. pseudosolanacearum* raised concerns about the emergence of additional RSSC species in Europe. Understanding the genetic diversity of RSSC strains present in mainland France, along with the structure and the dynamics of their population, is critical for maintaining effective epidemiological surveillance. In particular, MLVA is known to be a suitable molecular typing tool to trace bacterial introduction pathways, monitor pathogen spread at multiple geographical scales, and assess evolutionary potential. The insights provided by our study, with the development of a new MLVA scheme, are key to maintaining robust plant health surveillance and implementing targeted, evidence-based management strategies.

## INTRODUCTION

Globalization, the intensification of agriculture, and the resulting lack of cultivated plant diversity, as well as the current context of global climate change, have greatly favored the successive introductions and spread of plant pathogens, leading to an upsurge in outbreaks (1–3). In order to prevent and manage future epidemics, it is therefore necessary to characterize the incriminated plant pathogens, such as bacteria like those from the *Ralstonia solanacearum* species complex (RSSC), ranked as the second most threatening plant pathogenic bacteria in the world (4).

The RSSC is composed of three distinct species: *Ralstonia solanacearum*, *Ralstonia pseudosolanacearum*, and *Ralstonia syzygii* (5–8). Distributed worldwide, the RSSC infects more than 250 host plants (8–10), including many plants of major economic importance, such as potato or tomato. In view of its wide host range and ability to withstand a wide range of temperatures and ecoclimatic conditions, the RSSC is listed as a quarantine pest in the European Union, according to Commission Implementing Regulation (EU) 2019/2072 (11). Strains of the RSSC were first characterized into five races (12) on the basis of their host range, then into five biovars (13) on the basis of biochemical criteria, but these initial classifications lacked precision. Sequencing of the internal transcribed spacer region and the *hrpB* and *egl* genes has paved the way for a new one, with four phylotypes based on the geographical origin of the strains (I, Asia; II, America; III, Africa; and IV, Indonesia). Partial sequencing of the *egl* gene has made it possible to classify strains further into sequevars (6).

Groups of strains that are genetically distinct but adapted to identical or very similar ecological niches, or ecotypes, can also be distinguished within the RSSC. Among them, the brown rot ecotype includes strains of phylotype IIB, which damage potato crops. Several of them belong to sequevar 1, a group originating from South America and whose strains are known to be pathogenic in cold conditions, making them a major threat to temperate climates. In Europe, the first outbreaks were reported in Southern areas, in countries such as Italy, Greece, Portugal, and Spain, between 1922 and 1951 (14), then in Northern ones, with Sweden in 1972 (15). Later on and more specifically, the first major outbreaks of IIB-1 strains were confirmed in the early 1990s. Solanaceous crops such as potato and tomato were affected in several European countries on this occasion, including Belgium, the Netherlands, the United Kingdom, and France. The corresponding strains were also found in the water of several rivers (16). It is likely that a strain of IIB-1 arrived from South America at the beginning of the 20th century and then spread across Europe, hence initiating all the subsequent epidemics (17). Currently, *R. solanacearum* (phylotype IIB-1) is present in at least 15 of the 27 countries of the European Union, as shown in the European and Mediterranean Plant Protection Organization distribution map (18). Strains of this sequevar are known to be very similar genetically, even on a large geographical or temporal scale (17, 19–21). Parkinson et al. (22) speculate that this may be attributed to a relatively recent spread of this sequevar worldwide via contaminated plant material, which is also suggested by the work of Clarke et al. (17).

In France, the bacterium was first detected in 1994 on tomato, then in 1995 on greenhouse-grown and outdoor tomatoes, as well as in an experimental potato field (14). At the present time, *R. solanacearum* is still latently present in France, where the last outbreak was observed in 2017 on greenhouse-grown tomatoes in Maine-et-Loire (23), but the routes of invasion into mainland France have yet to be identified. Despite the importance and topicality of these issues, few epidemiological studies have been carried out on European strains of the RSSC (17, 22, 24) and none on French strains of the RSSC.

In addition, several reports of *R. pseudosolanacearum* have been made in Europe since 2015 (25). The first observations were made on contaminated rose plants in greenhouses in Belgium and the Netherlands (26), then on rose plants in greenhouses both in Poland (2017) and Switzerland (2018). From 2018 to 2024, *R. pseudosolanacearum* was reported in Germany, Greece, Hungary, Italy, the Netherlands, Poland, Slovenia, and Switzerland on several matrices: greenhouse-grown ginger, cucumber, tomato, and rose, as well as in tomato fields, nightshade, and irrigation water. These recent detections demonstrate the ability of *R. pseudosolanacearum* to succeed in a temperate climate and persist in the corresponding conditions for several years (27, 28). This also opens the door to the emergence of other species of the *Ralstonia* complex in Europe.

The multilocus variable-number tandem-repeat analysis (MLVA) is a powerful tool for investigating strain diversity and inferring the origin and pathways of bacterial invasion. The MLVA is a genotyping method based on the polymorphism of variable number of tandem repeats (VNTR). VNTRs are highly variable microsatellites (between 1 and 10 bp) or minisatellites (between 10 and 100 bp) consisting of a tandemly repeated pattern (29). By studying the number of repeats of several VNTRs within a population, it is possible to assign MLVA profiles, or haplotypes, to each individual, enabling them to be differentiated. This technique has been widely used on human and animal pathogens to trace their spread and to identify closely related variants. It was first used on a plant pathogen, *Xylella fastidiosa*, in 2001 (30). Other schemes soon followed for various plant pathogenic bacteria (31–35), including the RSSC with the MLVA schemes of N’guessan et al. (36) and Parkinson et al. (22). The latter, carried out on English strains of IIB-1, was used to identify the origin of several outbreaks that occurred near an infected river. Other MLVA schemes for different phylotypes of the RSSC appeared successively, including those of Ravelomanantsoa et al. (37), Guinard et al. (38), Ravelomanantsoa et al. (39), Rasoamanana et al. (40), and Cellier et al. (41). However, due to the near-clonal nature of the IIB-1 genetic group, additional VNTRs are needed to improve resolution and allow for more precise differentiation between strains from around the world.

The aims of this study were (i) to develop an MLVA scheme for differentiating strains of *R. solanacearum* belonging to the near-clonal IIB-1 sequevar; (ii) to describe the genetic diversity of closely related RSSC strains that have been collected in mainland France for nearly 30 years; and (iii) to investigate the invasion routes of those strains at local, national, and global scales.

## RESULTS

### VNTR screening led to the development of a 14-VNTR MLVA scheme

A total of seven VNTRs were selected from the literature after *in vitro* validation: L504, L539, L540, L563 (22), RS2BL21, RS2BL22, and RS2BL24, developed by N’guessan et al. (36) on phylotype II.

None of the VNTRs identified with Phobos were retained for the MLVA scheme as no diversity was observed. *In silico* screening for VNTR using TR Finder allowed the identification of 2,125 unique VNTRs in the genome of strain UW72. A subset of 128 VNTRs was chosen by selecting patterns that were at least 5 bp long, were repeated at least three times, and whose repeats matched at least at 95% with each other. After blasting this subset against the 260 genomes described previously, 11 of those 128 VNTRs were tested *in vitro*, as they were polymorphic and present in all genomes. Seven of them met the criteria set for *in silico* diversity and primer multiplexing: VMGP6, VMGP8, VMGP752, VCHR16, VCHR126, VCHR401, and VCHR1339.

The final MLVA scheme was composed of 14 VNTRs: 10 localized on the chromosome and 4 localized on the megaplasmid (Tables 1 and 2). VNTR sizes ranged from 6 to 15 bp, and patterns were repeated from 2 to 14 times within the 384 strains used in this study. In reference strain UW72, VNTR patterns were repeated from 2 to 12 times. The conservation of identical flanking regions for each VNTR was confirmed after sequencing of seven strains per VNTR with Sanger technology.

**TABLE 1:** Features of the selected VNTR and PCR conditions^*a*^

Primer name	Primer sequences (5'−3')	Annealing temperature (°C)	Multiplex number	Flanking region length (bp)	Pattern	VNTR size	Reference
L504	F-HEX-GTCGTTCGCGAAGTACGC	57	1	237	CTTGCCG/A	7	(2)
	R-CGATCAGCCCGAGCGATG						
L540	F-NED-GGCGTGATCCAGCGGTGCTT			317	TCGGTGAG	8	(2)
	R-CGGTGGTCTCTCGATGCAGAC						
RS2BL22	F-PET-CTCAGCGCATTCAAGCTGCC			168	GTAGCC	6	(3)
	R-GAGACCCAGCGGTTCGCTTC						
L563	F-FAM-GATGTGCTGACCGCCGAG	63	2	341	TCTAGCC	7	(2)
	R-CGTAGGCGTGGACAAGGCTG						
VMGP6	F-NED-CGTCGACCAGGTTCTCCG			410	GGCCGAAGG	9	This study
	R-GTGCGAAGGGCAAGCACTG						
VMGP8	F-VIC-GCAGCAGTTCCAGGTCGGC			214	GAGACGGCT	9	This study
	R-GCACAGGCACAGGCACAAG						
L539	F-FAM-CGATGCAGACCTCGATGGG	63	3	217	GCTGCCCTGCGCATT	15	(2)
	R-CGCCAGCGTCATCAGCGCTTG						
RS2BL21	F-PET-CGGCATGGAGGGTCGGGCTTGAGGTG			151	AGTGCCC	7	(3)
	R-CGTCATCAACAGCACCGCG						
RS2BL24	F-VIC-CGGGCGAAGGCTCGCAGGCCAA			314	AACATG	6	(3)
	R-GGCCGGACGATACATGCCACCGCTCAC						
VCHR16	F-NED-GGTAACGTGGTTCAGCGCG			295	ACCGGCACG	9	This study
	R-CAGTTGCTGGCAGCGGTTG						
VCHR401	F-FAM-CGCAATCGTTGCAGGAGCAGG	63	4	386	CGGCCCAGG	9	This study
	R-GAAGGTTTCCCAGTCGGCG						
VCHR1339	F-VIC-CGCGTCGATCAGGTTGCAG			330	GCCGCGC	7	This study
	R-CCTTCAAGGGCGAGATGCG						
VCHR126	F-FAM-GCCATGACCGACCATTCCG	63	5	189	GCGGGC	6	This study
	R-GAACGGGAACTTGCGTGCCAG						
VMGP752	F-NED-CACACTGATCGCCTCGGAAC			275	CGACAT	6	This study
	R-GGCTGCATGGGTTGGTCTC						

^*a*^ F, forward; R, reverse.

**TABLE 2:** Characteristics of the 14 VNTRs for the 384 global *R. solanacearum* IIB-1 strains used in this study^*a*^

VNTR locus	Range of repeat numbers	Total number of alleles	Start position on the genome of strain UW72 (bp)	Location	Number of repeats (strain UW72 as reference)	Hunter-Gaston index
L504	5–10	6	CHR-1375147	Hypothetical protein	7	0.273
L540	7–14	7	CHR-2398774	Intergenic region	11	0.181
RS2BL22	5–7	3	CHR-1550444	Salicylate synthase	6	0.279
L563	5–9	5	CHR-3265162	Intergenic region	5	0.148
VMGP6	3–6	4	MEG-74616	Maltotransferase domain-containing protein	4	0.155
VMGP8	3–4	2	MEG-150219	Type IV pilus assembly protein FimV	4	0.494
L539	3–6	4	CHR-1134468	Pseudouridine synthase	4	0.137
RS2BL21	6–12	7	CHR-2063807	Intergenic region	12	0.311
RS2BL24	9–10	2	MEG-1733152	Cobaltochelatase subunit CobN	9	0.090
VCHR16	2–3	2	CHR-1593208	Intergenic region	3	0.005
VCHR401	2–3	2	CHR-975612	Methyl-accepting chemotaxis protein	3	0.005
VCHR1339	2–3	2	CHR-3329526	Type II secretion system F family protein	2	0.005
VCHR126	2–3	2	CHR-302505	DUF1439 domain-containing protein	3	0.005
VMGP752	3–4	2	MEG-1900964	Intergenic region	3	0.005

^*a*^ Positions starting with “CHR-” indicate that the VNTR is located on the chromosome, and positions starting with “MEG-” indicate that the VNTR is located on the megaplasmid.

The Hunter-Gaston discriminatory index (HGDI) and allelic richness ranged from 0.005 to 0.494 and from 1 to 2, respectively (Table 2; Table S2). The most polymorphic VNTR was VMGP8 (HDGI = 0.494), followed by RS2BL21 (0.311), RS2BL22 (0.279), and L504 (0.273). Two VNTRs were monomorphic for all French strains (VCHR16 and VCHR401), and three VNTRs were monomorphic for non-French strains (VCHR126, VMGP752, and VCHR1339). Among all repeats available within VNTR L540’s repeat range (between 7 and 14 repeats), no strain displayed 13 repeats, indicating perhaps incomplete sampling in our collection.

**Fig 1:**
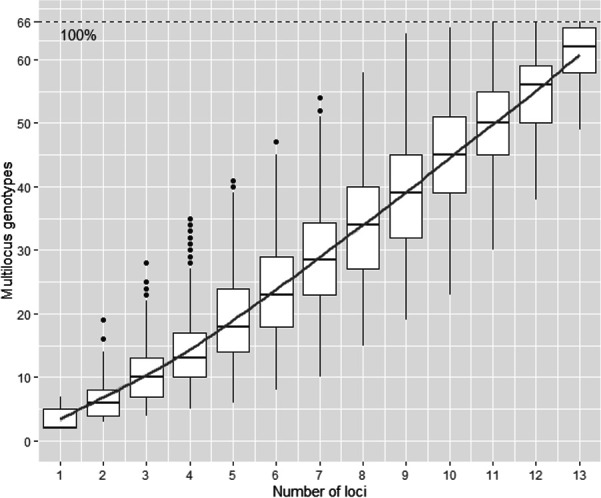
Genotypic accumulation curve (in percentage) of the 384 global *R. solanacearum* IIB-1 strains genotyped by the 14 VNTRs described in this study.

The genotypic accumulation curve (Fig. 1) showed a steady upward trend without reaching a clear plateau.

### Exploring the diversity of a worldwide collection of *R. solanacearum* strains

**Fig 2:**
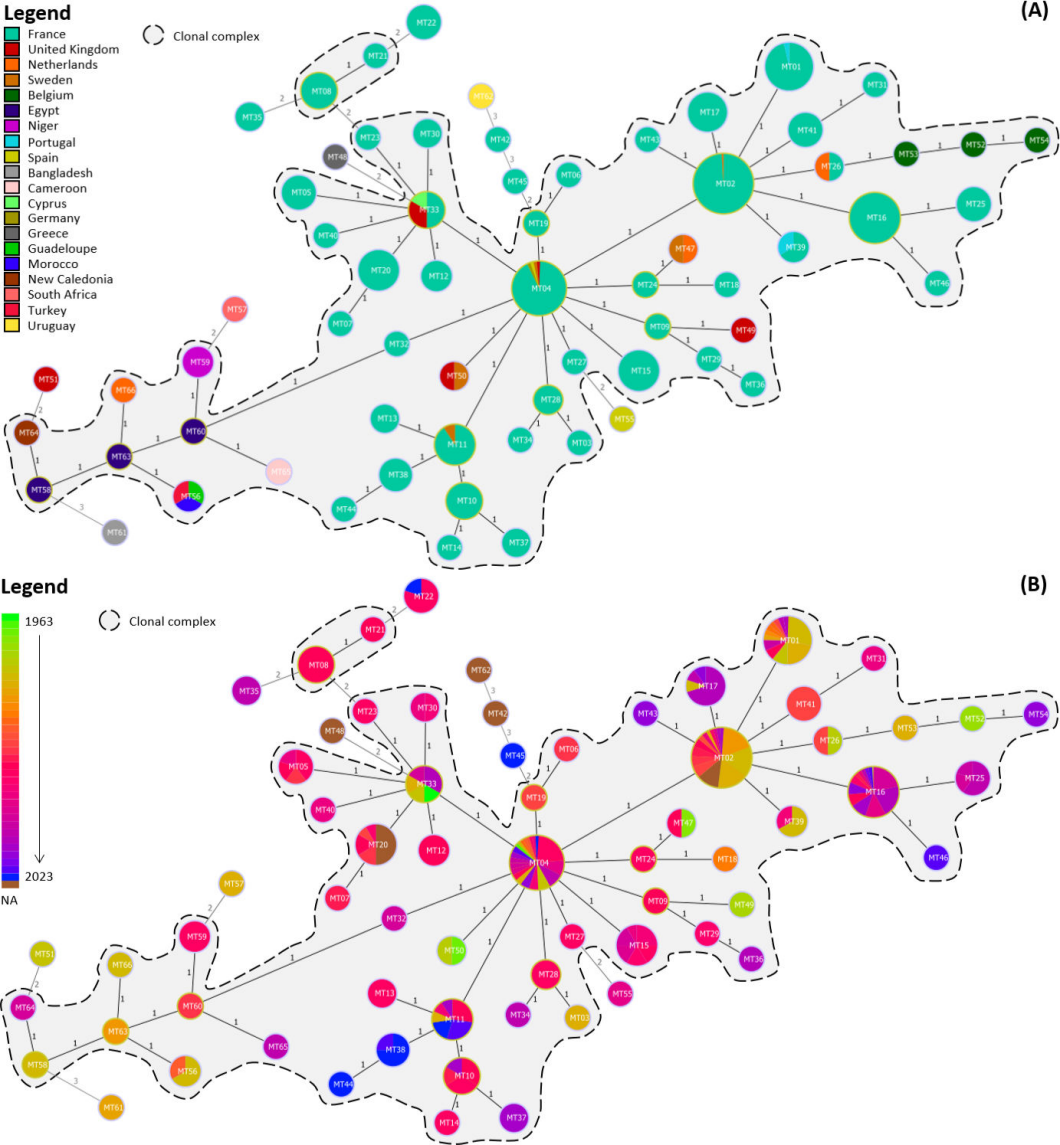
Minimum spanning trees built with the results of the typing of 384 global *R. solanacearum* IIB-1 strains using the 14 VNTRs described in this study. Haplotypes are colored according to (**A**) the country of origin and (**B**) the year of isolation. Each circle represents one haplotype, and circle sizes are not proportional to the number of strains belonging to the corresponding haplotypes, but they are not to scale. The number of loci differing between two haplotypes is represented by the distance, varying from one to three and displayed on each connecting line. Clonal complexes are underlined in gray. NA, not available.

A total of 66 haplotypes, named from MT01 to MT66, were identified within the 384 global strains (Fig. 2; Table S1). Most of the 66 haplotypes were closely related, mainly by single-locus variants (SLVs), while 18 haplotypes were linked by eight double-locus or three triple-locus variants. More specifically, the 346 French strains were characterized into 46 haplotypes.

Two clonal complexes were apparent on the minimum spanning tree (MST) (Fig. 2). The main clonal complex consisted of a group of 40 haplotypes, including French strains and another group of 14 strictly non-French haplotypes (MT47, MT49, MT50, MT52, MT53, MT54, MT56, MT58, MT59, MT60, MT63, MT64, MT65, and MT66), including strains from the Netherlands, Belgium, Portugal, the United Kingdom, Sweden, Spain, Germany, Cyprus, Niger, Egypt, Cameroon, Guadeloupe, Morocco, Turkey, and New Caledonia (Fig. 2A). The two most represented haplotypes were MT02 and MT04, which included 110 and 55 strains, representing 29% and 15% of the analyzed strain collection, respectively. A second smaller clonal complex included French haplotypes MT08 and MT21. Ten singletons of French (MT22, MT35, MT42, and MT45) or non-French (MT48, MT51, MT55, MT57, MT61, and MT62) origin were identified as well. Uruguayan strain LNPV 33.50 (MT62) was clearly distant from the other non-European strains as it was most closely linked to French haplotype MT42 by three locus variations. Strains represented by the two next most closely related haplotypes were both of French origin as well.

**Fig 3:**
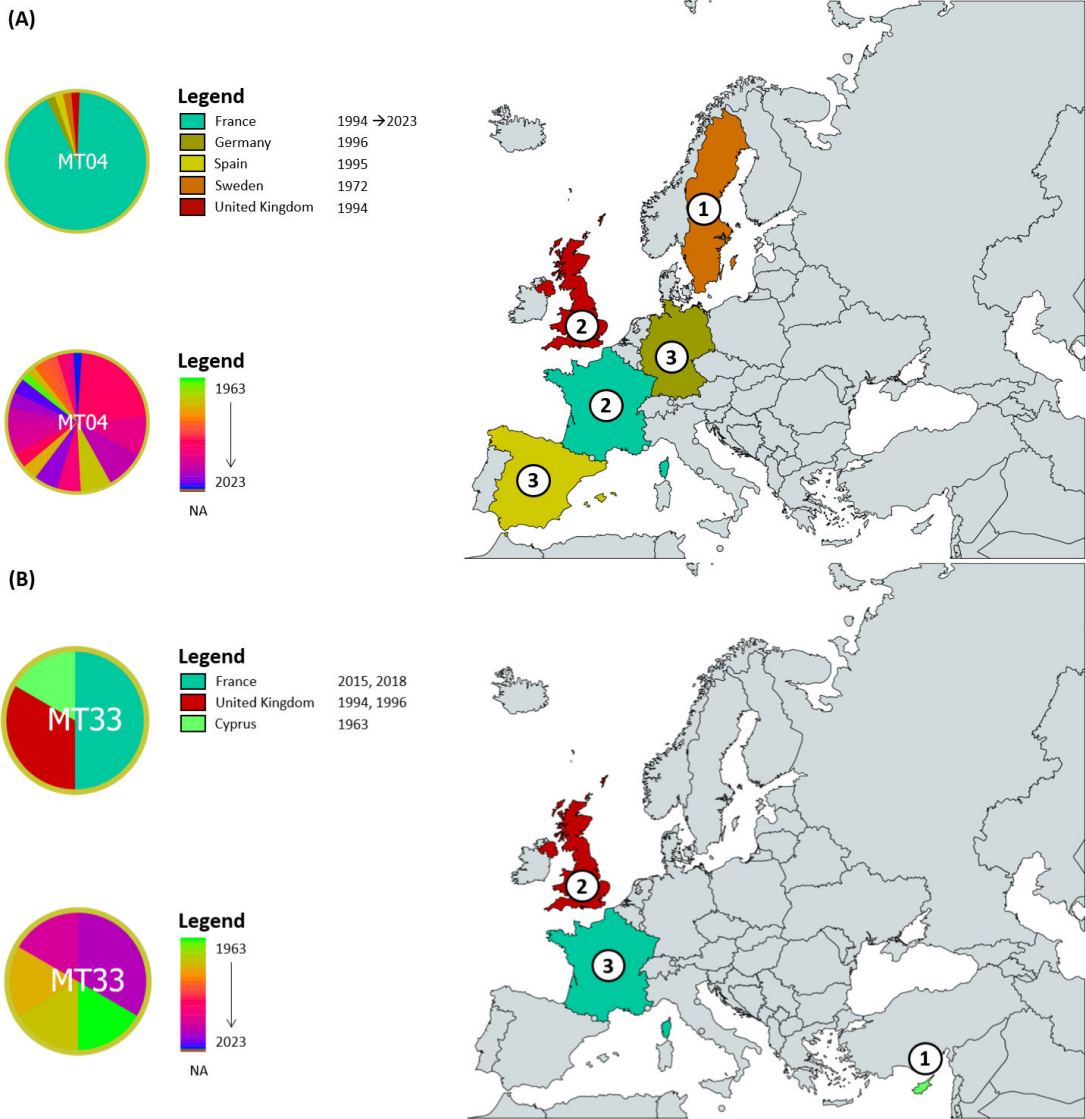
Sampling dates of strains belonging to (**A**) haplotype MT04 and (**B**) haplotype MT33, in regard to their country of isolation. Numbers indicate in chronological order the countries in which strains from haplotypes MT04 and MT33 were isolated. Map was created with MapChart.

No clear link was determined between the haplotype network structure and the years of isolation of the strains. Some haplotypes were characterized by strains isolated over restricted periods of time (MT05, MT15, MT25, MT38, and MT41), whereas haplotypes MT04, MT11, MT17, MT33, MT39, and MT47 displayed a high temporal variation in the isolation dates of their strains (Fig. 2B). Notably, MT04 included 55 strains isolated from 1972 (Sweden, *n* = 1) to 2023 (France, *n* = 1) (Fig. 3A); similarly, MT33 included six strains isolated over 55 years, from 1963 (Cyprus, *n* = 1) to 2018 (France, *n* = 2) (Fig. 3B). No explicit link could be identified between the haplotype and sample matrix of the strains either, mainly because of the limited number of strains within most haplotypes: more than half of the haplotypes (57%) were represented by a single strain (Fig. S1). Strains belonging to haplotype MT01 were recovered from more matrices than any other haplotype (tomato, water, nightshade, eggplant, and potato). Moreover, most strains were obtained in the framework of territory surveillance from water or nightshade samples which, while providing valuable insight into environmental reservoirs, may have influenced strain representation.

### Discriminant analysis of principal components reflects the global diversity revealed by the 14-VNTR MLVA scheme

**Fig 4:**
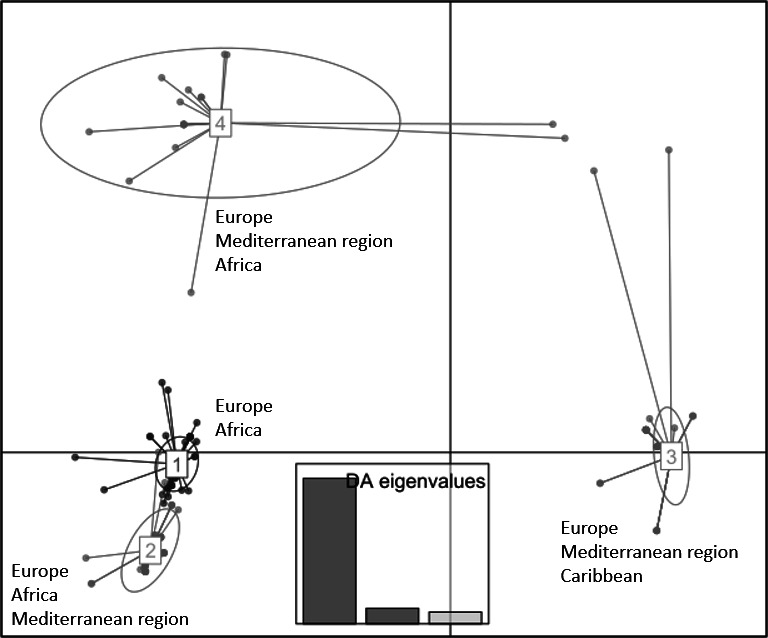
Scatterplot representing the clusters inferred by the discriminant analysis of principal components for the 384 global *R. solanacearum* IIB-1 strains investigated in this study. The eigenvalues indicated that the genetic structure was effectively represented by the first three principal components. The strains are represented by the dots. DA, discriminant analysis.

A discriminant analysis of principal components (DAPC) was performed on the 384 strains of the working collection and led to the identification of four clusters (Fig. 4; Fig. S2; Table S1). The eigenvalues revealed that the genetic structure was effectively represented by the first three principal components. Clusters 1, 2, and 3 were structured along the first axis. In particular, clusters 1 and 2 gathered strains isolated from Europe/the Mediterranean region and Africa. Cluster 3, distinctly separated on the horizontal axis, regrouped strains from Europe/the Mediterranean region and one strain from the Caribbean. Cluster 4 was separated from clusters 1 and 2 by the vertical axis and displayed more intra-cluster variability than the other clusters by gathering strains from Europe, Asia, South America, and Oceania. In addition, DAPC clusters 1, 2, and 3 regrouped haplotypes that were mostly closely related on the MST. In particular, cluster 1 gathered haplotypes that were linked by SLV only. However, no obvious relationship could be determined between the DAPC clusters and the country of origin or the year of isolation (Fig. S2), or the sample matrix (data not shown).

### Exploring the diversity of *R. solanacearum* strains at local scales

**Fig 5:**
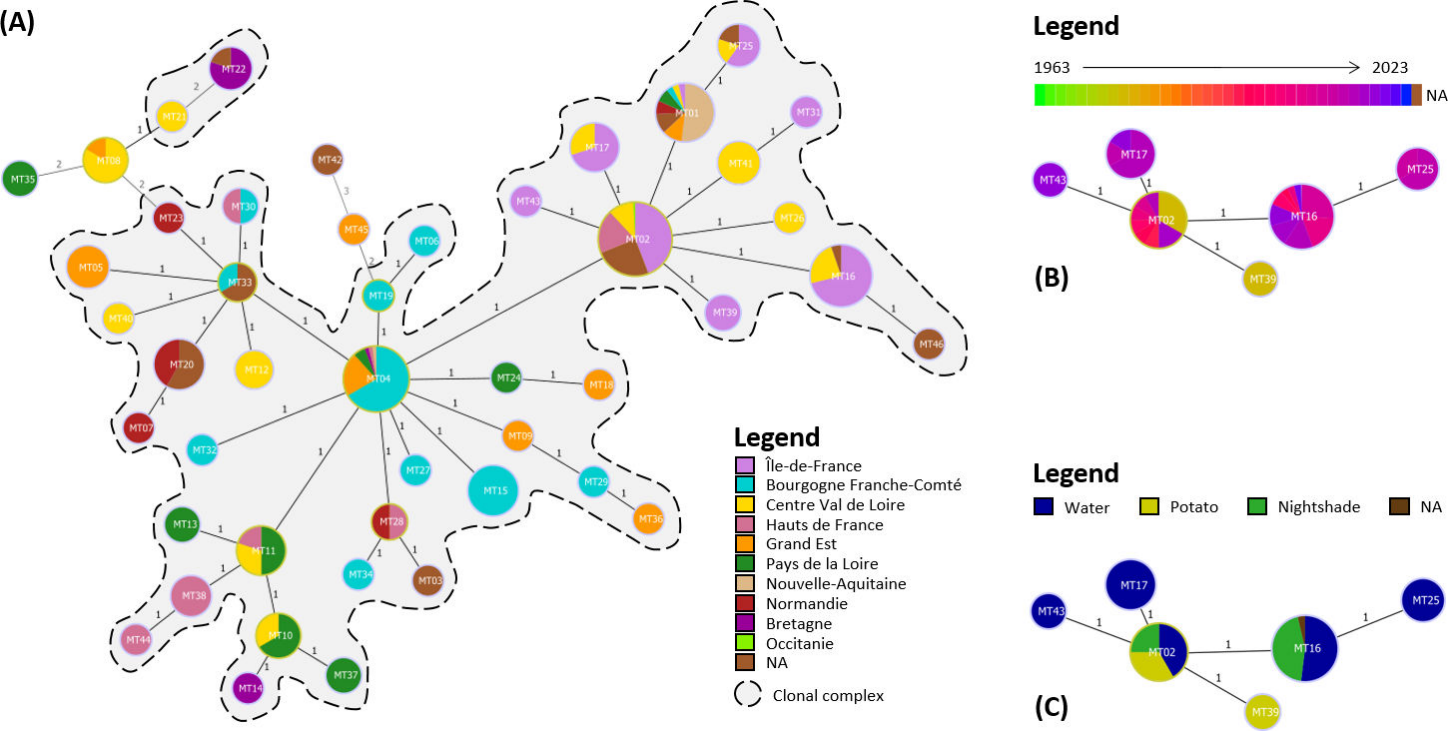
Minimum spanning trees built with the typing results using the 14 VNTR of (**A**) the 346 French *R. solanacearum* IIB-1 strains, with haplotypes colored according to the region of origin of the strains; (**B**) the 50 Essonne strains, with haplotypes colored according to the isolation year; and (**C**) the 50 Essonne strains, with haplotypes colored according to the sample matrix of the strain.

Forty-seven strains collected in the Center Val de Loire region over 15 years (2004–2019) on bell pepper, nightshade, potato, tomato, and water were characterized into 13 haplotypes differing by one to five loci (Fig. 5A). No clear link could be established between the MST structure and most of the French regions. On the other hand, haplotype networks of the set of 50 strains isolated during sampling campaigns which have been carried out in the locality of Essonne (France) every year since 1995 were also analyzed separately (Fig. 5B and C). Six haplotypes were discriminated among the 50 strains and were closely related by SLV. Most of the Essonne strains belonged to haplotypes MT16 and MT02 (54% [*n* = 27] and 24% [*n* = 12]), respectively. In particular, despite representing only 13% of the collection used in this study, strains from Essonne accounted for 71% of those belonging to haplotype MT16.

## DISCUSSION

Isolations of *R. solanacearum* over a 30-year period in mainland France resulted in a collection of 346 strains. This extensive collection provided a unique opportunity to characterize its genetic diversity and gain deeper insights into the origins of outbreaks in mainland France. MLVA has previously demonstrated its efficiency for establishing the source of sporadic epidemics caused by bacterial pathogens, such as the human and animal pathogens *Brucella* spp., for example (42–45). This technique has also been proven to be relevant for differentiating between strains of phytopathogenic bacteria collected over very restricted time periods (39, 46) or geographical areas (47), including strains collected on the same plant (48). In addition, MLVA represents a simple, rapid-to-implement, and cost-effective method, and we therefore applied it to differentiate the strains in our collection.

A novel MLVA scheme including 14 VNTRs, sourced from literature or developed in this study, was used to differentiate between strains of *R. solanacearum* belonging to the IIB-1 sequevar. All studies aiming to describe the genetic diversity of IIB-1 populations (39, 49–52) were based on the VNTR previously described by Parkinson et al. (22) and N’guessan et al. (36) and established the near-clonal nature of this sequevar. Parkinson et al. (22) and Ravelomanantsoa et al. (39) concluded that their MLVA schemes were highly discriminatory in view of the limited genetic diversity of IIB-1 strains. In particular, Parkinson et al. (22) identified only 10 haplotypes within 60 strains isolated in England between 1992 and 2009 (on average 6 strains per haplotype), whereas Ravelomanantsoa et al. (39) identified only 31 haplotypes within 255 strains isolated in Madagascar in 2013 (on average 8 strains per haplotype). This is consistent with our results, as we identified only 46 haplotypes within 346 strains isolated in mainland France (on average 7 strains per haplotype).

For the first time since the publication of Parkinson et al. (22), seven novel IIB-1-specific VNTRs were identified. The inclusion of those VNTRs to seven already described VNTRs allowed the differentiation of 14 additional haplotypes from mainland France. All repeat variations consisted of single-repeat changes, with the exception of VNTR L540, for which no strain displaying 13 repeats of the pattern was present in the data set. However, since no variation of three or more repeats was observed within the 14 VNTRs used in this study, it can be considered that all VNTRs followed stepwise mutations (53). In particular, VMGP8 was the most discriminatory VNTR (HGDI = 0.494). However, the relatively low values of the HGDI overall highlighted the reduced genetic diversity within the strains that were investigated.

We investigated the discriminatory power of 536 VNTRs identified by two distinct algorithms, and only 1.3% (*n* = 7) were found to be useful for distinguishing the IIB-1 strains investigated in our study, underlining the scarceness of relevant markers allowing us to differentiate between IIB-1 strains. This might indicate, in addition to the limited discriminatory power of several VNTR (VCHR16, VCHR126, VCHR401, VMGP752, and VCHR1339), that the search for new IIB-1-specific VNTR is becoming increasingly challenging as well. Most of the 66 haplotypes described in this work were closely linked by SLV, which corroborates the highly clonal nature of *R. solanacearum* IIB-1 strains, even in a large collection of strains with high temporal and geographical diversity, and confirms that little diversification occurs over time within this genetic group (17). Additionally, although the absence of a plateau in the accumulation curve suggests that some genetic diversity may remain undetected, the combined analysis with MST and haplotype data still supports the conclusion of limited genetic diversity among the analyzed strains.

A subgroup of non-French strains was clearly separated on the MST (including MT51, MT56, MT57, MT58, MT59, MT60, MT61, MT63, MT64, MT65, and MT66). This cluster included at least one strain from each continent and, in particular, all African strains (*n* = 9), linked by SLV. Between 1994 and 1998, strains were isolated in several countries: the United Kingdom (MT51) in 1994, the Netherlands (MT66), Egypt (MT58), Morocco and Turkey (MT56) in 1995, then South Africa in 1996 (MT57), Bangladesh in 1997 (MT61), and Egypt in 1998 (MT63). Those observations suggest that European and African strains are closely related, a hypothesis which is supported by previously published articles (17, 54, 55) in which a Mediterranean origin for the European strains is most frequently suspected, especially in light of the potato trade from Malta, Cyprus, and Egypt to western Europe in the 1960s and 1970 s (56).

Such investigations can be conducted on a European scale as well. Within the strains used in this study, four have been isolated in Europe prior to 1990, which is commonly acknowledged as the beginning of the European outbreaks (17, 54, 57). Those four strains correspond to different haplotypes, namely MT04, MT47, MT50, and MT52. This suggests either that (i) the first strains introduced in Europe already displayed some genetic diversity and that the European outbreaks reported in the 1990 s were induced by several strains of distinct genetic lineages, rather than by a single clone, or (ii) a single strain was first introduced in the Middle East or Europe several years prior to the European outbreaks and its descendants diverged into several haplotypes. The latter hypothesis seems to be supported by the works of Janse (14, 56), who highlights the rise of potato imports from the Middle East and North Africa into Europe starting in the 1960s, which led to an increased number of brown rot interceptions in several European countries. The discrimination of 98 European strains sampled between 1990 and 1999 into 14 haplotypes (MT01, MT02, MT03, MT04, MT11, MT17, MT26, MT33, MT39, MT49, MT50, MT51, MT53, MT66) strengthens this hypothesis as well, as it indicates that strains originated from diverse genotypic backgrounds since the first European outbreaks. In addition, the persistence over the years of MT33 implies that strains introduced in Europe prior to or during the 1990 s outbreaks were able to subsist in this area over several years.

To explore further the origin of the European outbreaks, potential dispersion routes can be inferred based on the isolation dates and countries of origin of the investigated strains. For instance, MT04 likely originated from an initial European introduction in Sweden before 1972, which Persson (15) speculates might originate from a potato processing plant located nearby a river stream. Strains may then have been introduced to the United Kingdom, France, Germany, and Spain during the European outbreaks of 1994 and 1995, which were characterized by particularly hot summers (58, 59) likely facilitating locally the establishment of *R. solanacearum* across several countries (Fig. 3A). Similarly, MT33 strains appear to have emerged in Cyprus in 1963, later spreading to the United Kingdom during the European epidemics and, more recently, to France (2015 and 2018) (Fig. 3B).

Here, the use of MLVA allowed the discrimination of 346 French strains from one genetic group, sequevar IIB-1, into 46 haplotypes. Some haplotypes seem to have persisted in France since the earliest outbreaks, such as MT02 and MT04, which had a star-like structure on the MST (Fig. 2), as several haplotypes differing only by SLV were connected to them. This star-like structure is characteristic of outbreaks from founder haplotypes (39), particularly given that both haplotypes are shared by a large number of strains. Regarding these results, MT02 and MT04 might be identified as potential founder haplotypes of the first French outbreaks. Besides, despite being the most represented haplotype in the collection used in this study, only one strain represented by MT02 (<1%) was of non-French origin (the Netherlands), which suggests that this haplotype mostly spread in France. In addition, some very recent haplotypes have been described (MT37, MT38, MT43, MT44, MT45, MT46, MT54), which suggests either (i) a single introduction when French outbreaks were initiated and from which new haplotypes emerged, or (ii) distinct introductions of closely related *R. solanacearum* strains in France, possibly from other locations in Europe and the Mediterranean area, considering that several haplotypes (MT01, MT02, MT04, MT11, MT26, MT33, and MT39) are shared between France and other European/Mediterranean countries. This would be consistent with the works of Clarke et al. (17) which identified frequent *R. solanacearum* strains occurrences between Northern Europe, the Mediterranean region, and Africa. Similarly, Caruso et al. (60) determined that several introductions of *R. solanacearum* were likely to have occurred in Spain.

This can be attributed both to the anthropogenic activities (such as the trade of contaminated plant material and non-compliance with prophylactic measures) which facilitate the global dissemination of IIB-1 strains; and also to the geographical proximity of European countries, which allows for strain dissemination through natural pathways such as watercourses. It is particularly plausible to make this assumption in light of the current spread of *R. pseudosolanacearum* in Europe, some corresponding strains having been isolated in irrigation water and several imported crops (28, 61).

MLVA schemes have previously been developed for investigating the diversity of strains collected on the same field (38), demonstrating the ability of this method to discriminate strains isolated on a restricted geographical area. Similarly, the MLVA scheme described in this study proved useful for distinguishing the 50 strains collected in the French Essonne ‘département’ (i.e., county) over 29 years, differentiating them into six haplotypes. In particular, the five oldest Essonne strains (isolated in 1995) belonged to haplotypes MT02 (*n* = 4) and MT39 (*n* = 1), which may have been the first ones introduced in Essonne. Haplotypes MT16, MT17, MT25, and MT43 likely emerged over time, while haplotype MT02 persisted in the locality until at least 2019. This might suggest that a single introduction occurred in Essonne, which would account for the low genetic diversity of the strains isolated in this locality. In contrast, the Centre-Val de Loire region harbors the highest genetic diversity of *R. solanacearum* strains in France (Fig. 5A), with 47 strains isolated between 2004 and 2019 characterized into 13 haplotypes. This higher genetic diversity may be attributed to multiple introductions into the region over time. Alternatively, host pressure or bottleneck effect could be responsible for a heightened genetic diversity.

By suggesting that the genetic diversity of *R. solanacearum* strains varies according to the isolation region in mainland France, our data indicate that different evolutionary histories may have taken place at different geographical scales on French territory. It also implies that our MLVA scheme is fully relevant to discriminate French IIB-1 *R. solanacearum* strains, as well as global IIB-1 *R. solanacearum* strains.

### Conclusion

MLVA has been repeatedly proven to be a useful tool for distinguishing closely related strains even when studying bacteria such as *Yersinia pestis* (62, 63) and *Mycobacterium tuberculosis* (64, 65), widely known for their highly clonal origin. Here, we developed an MLVA scheme specific to monomorphic IIB-1 *R. solanacearum* strains. Our scheme proved useful for differentiating strains at global, national, and local scales, demonstrating its relevance for reconstructing the invasion pathways of IIB-1 strains throughout the world. Additionally, our publicly available data (Table S1) can be used by the scientific community to generate haplotype MST, allowing integration of additional IIB-1 strains for extended epidemiological analyses. It would be valuable for future research to use this MLVA scheme for typing more recent global strains and acquire further understanding of the global dispersal and invasion routes of sequevar IIB-1.

## MATERIALS AND METHODS

### Bacterial strains

A total of 384 strains were used in this study: 346 strains from mainland France and 38 non-French strains (Table S1). French strains were provided by the Laboratoire de la Santé des Végétaux (ANSES, Angers, France) and the Agricultural Technical Institute for Seed Potato (inov3PT, Paris, France). Non-French strains were sourced from ANSES or inov3PT as they were maintained in their respective collections or from international collections. Isolations were carried out following the different steps described in PM7/21 (2022), from different matrices (water, nightshade, potato, tomato, eggplant, bell pepper, and *Pelargonium*) between 1994 and 2023 in mainland France as part of territory surveillance or sampling campaigns. Bacteria were stored in peptone glycerol broth (prepared by dissolving 5 g of yeast extract and 5 g of peptone in 1 L of demineralized water and 150 mL of glycerol) at −80°C.

### Phylotype and sequevar identification

RSSC phylotypes were assigned to the strains using the phylotype-specific multiplex-PCR primer pairs described in Fegan and Prior (6) and the 759 out of 760 primer pair described in Opina et al. (66). All PCRs were carried out using the Platinum Taq DNA Polymerase kit (Thermo Fisher, USA). Phylotypes were identified based on the size of PCR products revealed on a 1.5% agarose gel. The sequevar was identified using the Endo-F/Endo-R primer pair described in Fegan and Prior (6) amplifying the partial sequence of the *egl* gene. PCR products were purified and sequenced by Eurofins (Eurofins Genomics, Koeln, Germany) using Sanger technology. A maximum likelihood phylogenetic tree was built with the sequenced amplicon and 176 other *egl* sequences belonging to all previously described sequevars, using the PhyML plugin (67) from Geneious Prime 2022.0.2 software (Biomatters) and as described by Cellier et al. (68). Sequevars were attributed to the strains according to the cluster they belonged to.

### VNTR selection from literature

Preliminary experiments were carried out to check for interest of VNTR specifically targeting phylotype II or IIB-1 already described in previous works. Five VNTRs described by Parkinson et al. (22) (L504, L539, L540, L563, and L578) and 12 VNTRs described by N’guessan et al. (36) (RS2AL01, RS2AL02, RS2AL03, RS2AL04, RS1L05, RS3L17, RS3L19, RS2BL21, RS2BL22, RS2BL23, RS2BL24, and RS2BL25) were tested on a set of seven strains representative of the diversity (host plant, year of isolation, and geographical location) of the French collection (LNPV 14.19, LNPV 20.10, LNPV 26.18, LNPV 34.02, LSV 42.17, LSV 48.68, and LSV 52.17). Strains were plated on semi-selective medium yeast peptone glucose agar (YPGA) (69) and grown at 28°C for 48–72 h. One isolated colony was selected, grown on a new YPGA plate for 24 h at 28°C, and a 1 µL loop of bacterial material was diluted in 200 µL of ultra-pure water to be used as template for PCR. The PCR was performed using Taq Platinum (Invitrogen, USA) and the following conditions: an initial denaturation step at 94°C for 2 min, 30 cycles of denaturation at 95°C for 30 s, annealing at a specific temperature (Table 1) for 30 s and extension at 72°C for 1 min, and a final extension step at 72°C for 10 min. PCR products were revealed on a 1.5% agarose gel stained in an ethidium bromide bath to validate the PCR amplification and were later sequenced by Eurofins (Eurofins Genomics) with Sanger technology in order to check the number of pattern repeats and that the length of the flanking regions remained the same.

### Identification of novel VNTR

As the first step, the publicly available genome sequences of phylotype IIB-1 strains CFIA906, IPO1609, UW551, UY031, and CFBP3858 were screened for the presence of VNTR using the Geneious Prime plugin Phobos (70). The following criteria were settled: perfect search, repeat unit length between 5 and 100 bp, typical analysis, minimum length at 10 bp (or minimum two repeats), minimum score at 6, maximum 0f successive N’s, and N’s treated as neutral. Primers were designed for the VNTR, which met the following criteria: (i) *in silico* diversity was observed between the five genomes used for the screening, and (ii) the pattern was repeated at least twice in all five genomes.

Additionally, the genome sequence of strain UW72 (GCF_021117095.1) was screened for VNTR using Tandem Repeat Finder (71). The following criteria were settled in the advanced options: alignment parameters of 2,7,7; minimum alignment score of 80; maximum period size of 500 bp; maximum TR array of 2 Mb; minimum unit length of 4 bp; and minimum of two repeats. Candidate VNTRs were then blasted on 235 French and four European non-French genomes from our collection, and 21 public European non-French genomes. Primer pairs were designed and used for amplification whenever *in silico* diversity was observed among the tested strains (Table 1).

For all primers, a maximum size of 500 bp was set for all amplicons. The primers were designed to allow multiplexing whenever possible, based on the hybridization temperature of each primer pair, the estimated size of the PCR products, and the absence of cross-dimers between primers.

### *In vitro* validation of the MLVA scheme

VNTRs were kept when a difference of at least one repeat was visible among the amplified sequences of the tested strains after Sanger sequencing. Primers marked with fluorophores were then used for amplification of each VNTR within the strain collection of French and non-French strains (Table 1). All final PCR products were revealed on a 1.5% agarose gel stained in an ethidium bromide bath before undergoing microsatellite genotyping using capillary electrophoresis (Applied Biosystems, USA) at the Gentyane Platform.

### Data analysis

The number of repeats of each VNTR was determined based on the following formula: (total size of the amplicon – size of the flanking regions) / pattern repeat size. All nucleotide sequences were visualized and manipulated using Geneious Prime 2022.0.2 software (Biomatters). MSTs were constructed with Phyloviz (72) using the recommended algorithm for analysis of MLVA data, combining global optimal eBURST and Euclidean distances. Clonal complexes were defined as haplotypes that differed from one another by only one locus. The HGDI (73), based on the probability of two unrelated strains being characterized as the same type, was used to assess the discriminatory power of each VNTR.

An assessment of the population structure was conducted using DAPC, a clustering method independent of population genetic models. The clustering algorithm *k*-means was run with increasing values of *k*, in order to identify the optimal number of clusters which maximizes between-groups variation. This usually corresponds to the lowest Bayesian information criterion value. Thirty independent *k*-means runs were conducted to evaluate the clustering stability. Analyses were performed using the “adegenet” package in R (74). The genotype accumulation curve was obtained using the “poppr” package in R (75).

## SUPPLEMENTAL MATERIAL

Supplemental material aem.00963-25-s0001.docx Table S2; Fig. S1 and S2.

Table S1 aem.00963-25-s0002.xlsx *Ralstonia solanacearum* strains used in this study, with corresponding metadata and genotypic data.

ASM does not own the copyrights to Supplemental Material that may be linked to, or accessed through, an article. The authors have granted ASM a non-exclusive, world-wide license to publish the Supplemental Material files. Please contact the corresponding author directly for reuse.
