# Supplementary material for: Exploring the diversity of Ralstonia solanacearum strains from mainland France using a novel multiple loci VNTR analysis scheme
Source: Appl Environ Microbiol. 2025 Jul 31;91(8):e00963-25. doi: 10.1128/aem.00963-25 (PMC12366337; doi:10.1128/aem.00963-25)
Supplement: Supplemental material — Table S2; Fig. S1 and S2. [file aem.00963-25-s0001.docx]

**Table S1**: see separate .xlsx “Table_S1.xlsx”.

**Table S2**: allelic richness of the 14 VNTR according to the country of isolation of the strains.

|  | **Bangladesh** | **Belgium** | **Cameroon** | **Cyprus** | **Egypt** | **France** | **Germany** | **Greece** | **Guadeloupe** | **Morocco** | **Netherlands** | **New-Caledonia** | **Niger** | **Portugal** | **South Africa** | **Spain** | **Sweden** | **Turkey** | **United Kingdom** | **Uruguay** |
| --- | --- | --- | --- | --- | --- | --- | --- | --- | --- | --- | --- | --- | --- | --- | --- | --- | --- | --- | --- | --- |
| **L504** | 2 | 1.73 | 2 | 2 | 1.60 | 1.57 | 2 | 2 | 2 | 2 | 1.68 | 2 | 1.60 | 1.83 | 2 | 1.67 | 1.68 | 2 | 1.55 | 2 |
| **L540** | 1 | 1.00 | 1 | 1 | 1.00 | 1.13 | 1 | 1 | 1 | 1 | 1.00 | 1 | 1.00 | 1.00 | 2 | 1.50 | 1.00 | 1 | 1.17 | 1 |
| **RS2BL21** | 2 | 1.00 | 2 | 2 | 1.73 | 1.15 | 1 | 2 | 2 | 2 | 1.46 | 2 | 1.60 | 1.00 | 2 | 1.00 | 1.00 | 2 | 1.41 | 2 |
| **RS2BL22** | 2 | 1.60 | 2 | 2 | 1.60 | 1.57 | 2 | 2 | 2 | 2 | 1.68 | 2 | 1.60 | 1.67 | 2 | 1.67 | 1.57 | 2 | 1.62 | 2 |
| **L539** | 2 | 1.73 | 2 | 2 | 1.73 | 1.53 | 2 | 2 | 2 | 2 | 1.57 | 2 | 1.60 | 1.67 | 2 | 1.67 | 1.68 | 2 | 1.67 | 2 |
| **L563** | 2 | 1.60 | 2 | 2 | 1.60 | 1.53 | 2 | 2 | 2 | 2 | 1.68 | 2 | 1.60 | 1.67 | 2 | 1.83 | 1.57 | 2 | 1.68 | 2 |
| **VMGP8** | 2 | 1.60 | 2 | 2 | 1.60 | 1.62 | 2 | 2 | 2 | 2 | 1.71 | 2 | 1.60 | 1.67 | 2 | 1.67 | 1.57 | 2 | 1.55 | 2 |
| **VMGP6** | 2 | 1.60 | 2 | 2 | 1.60 | 1.54 | 2 | 2 | 2 | 2 | 1.57 | 2 | 1.60 | 1.67 | 2 | 1.67 | 1.68 | 2 | 1.55 | 2 |
| **RS2BL24** | 2 | 1.60 | 2 | 2 | 1.60 | 1.52 | 2 | 2 | 2 | 2 | 1.57 | 2 | 1.60 | 1.83 | 2 | 1.67 | 1.57 | 2 | 1.55 | 2 |
| **VCHR16** | 2 | 1.60 | 2 | 2 | 1.60 | 1.50 | 2 | 2 | 2 | 2 | 1.57 | 2 | 1.60 | 1.67 | 2 | 1.67 | 1.57 | 2 | 1.55 | 2 |
| **VCHR126** | 2 | 1.60 | 2 | 2 | 1.60 | 1.50 | 2 | 2 | 2 | 2 | 1.57 | 2 | 1.60 | 1.67 | 2 | 1.67 | 1.57 | 2 | 1.55 | 2 |
| **VMGP752** | 2 | 1.60 | 2 | 2 | 1.60 | 1.50 | 2 | 2 | 2 | 2 | 1.57 | 2 | 1.60 | 1.67 | 2 | 1.67 | 1.57 | 2 | 1.55 | 2 |
| **VCHR1339** | 2 | 1.60 | 2 | 2 | 1.60 | 1.50 | 2 | 2 | 2 | 2 | 1.57 | 2 | 1.60 | 1.67 | 2 | 1.67 | 1.57 | 2 | 1.55 | 2 |
| **VCHR401** | 2 | 1.60 | 2 | 2 | 1.60 | 1.50 | 2 | 2 | 2 | 2 | 1.57 | 2 | 1.60 | 1.67 | 2 | 1.83 | 1.57 | 2 | 1.55 | 2 |


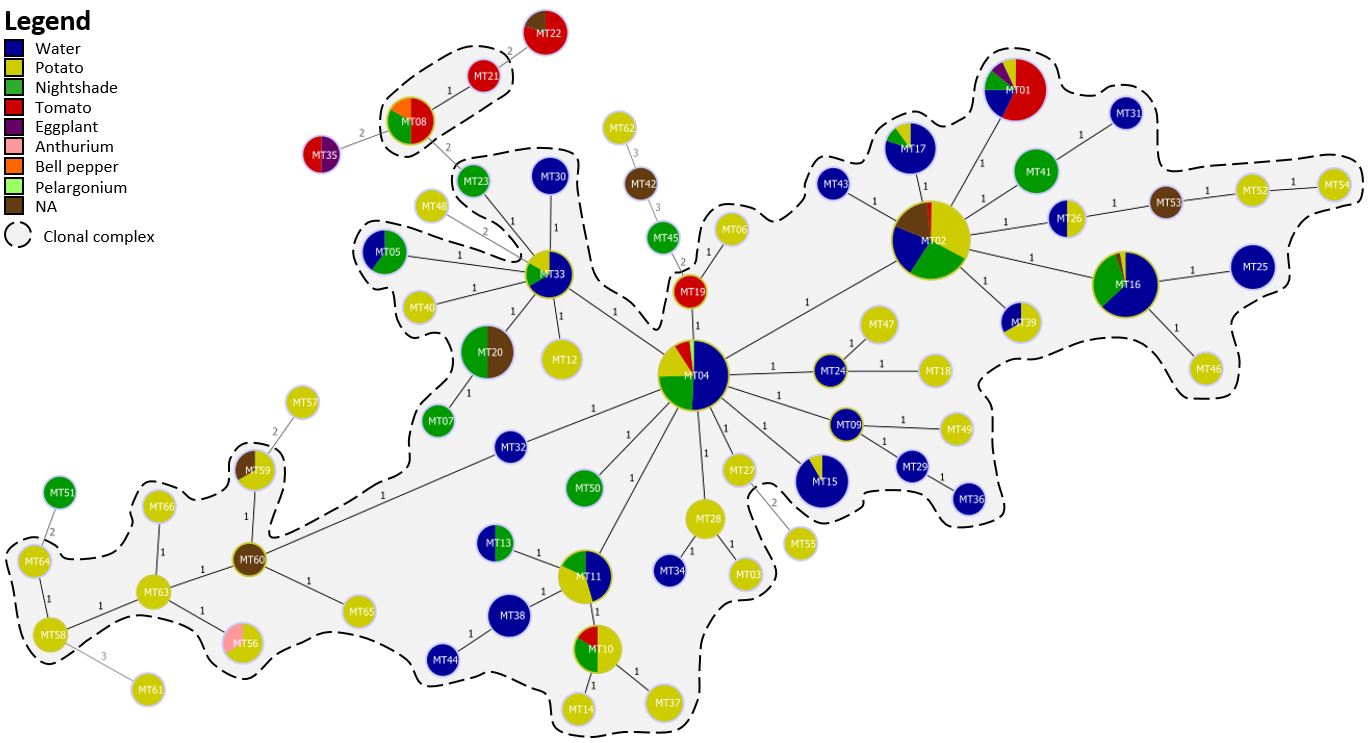


**Figure S1**: Minimum spanning tree (MST) of the 384 global *R. solanacearum* IIB-1 strains typed using the 14 VNTR described in this study. Haplotypes are colored according to the host of isolation of the strains. Each circle represents one haplotype, and **circle sizes are not proportional to the number of strains belonging to the corresponding haplotypes but they are not to scale**. The number of loci differing between two haplotypes is represented by the distance, varying from 1 to 3. Clonal complexes are underlined in gray.

**
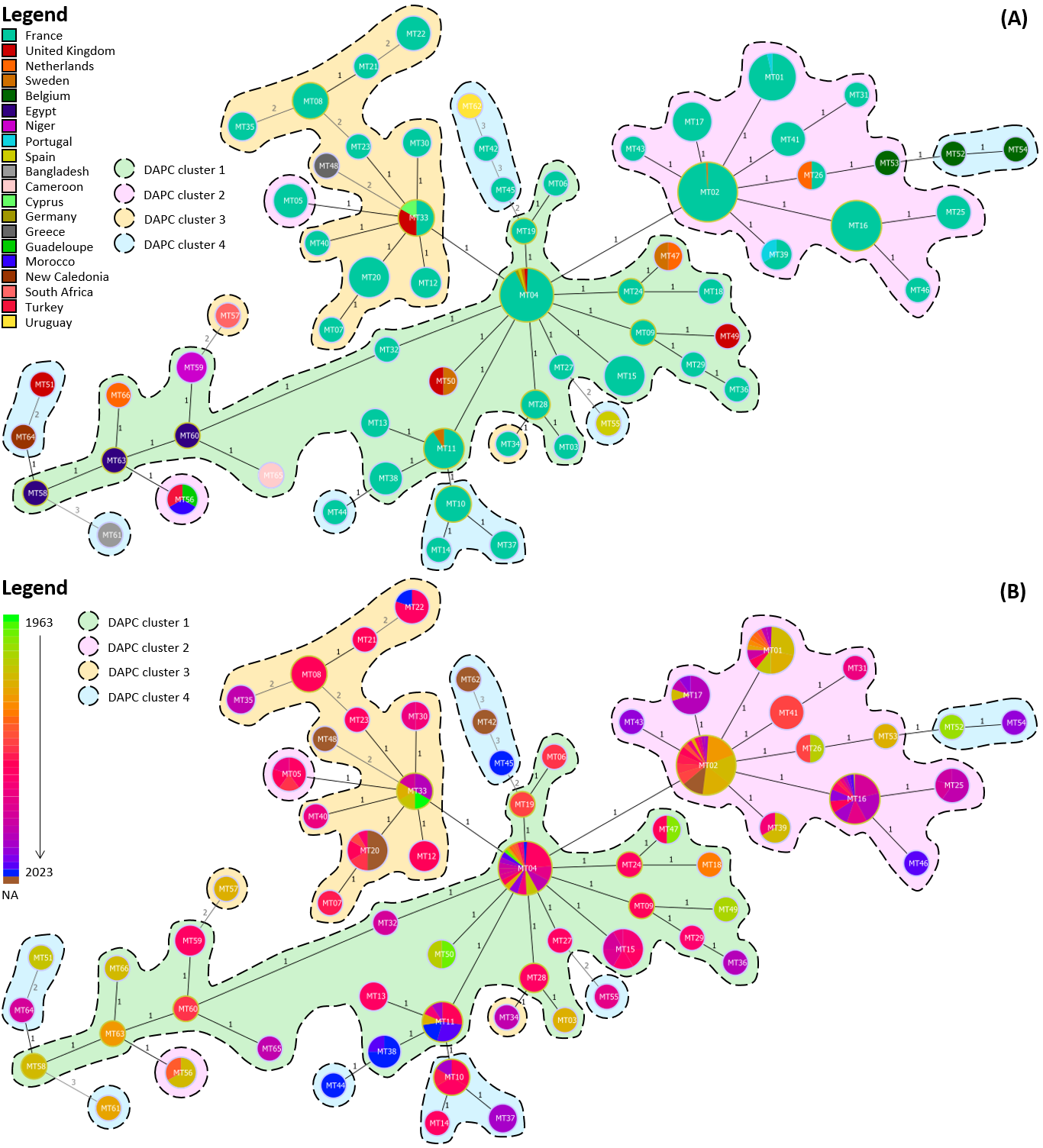
**

**Figure S2**: Minimum spanning trees (MST) of the 384 global *R. solanacearum* IIB-1 strains typed using the 14 VNTR described in this study. Haplotypes are colored according to (A) the country of origin; (B) the year of isolation. Each circle represents one haplotype**. Circle sizes are proportional to the number of strains belonging to the corresponding haplotypes but are not to scale but they are not to scale**. The number of loci differing between two haplotypes is represented by the distance, varying from 1 to 3. DAPC clusters are underlined in color.
